# Measuring Spectral Inconsistency of Multispectral Images for Detection and Segmentation of Retinal Degenerative Changes

**DOI:** 10.1038/s41598-017-11730-y

**Published:** 2017-09-12

**Authors:** Jian Lian, Yuanjie Zheng, Peiyong Duan, Wanzhen Jiao, Bojun Zhao, Yanju Ren, Dinggang Shen

**Affiliations:** 1 Shandong Normal University, School of Information Science and Engineering, Key Lab of Intelligent Computing & Information Security in Universities of Shandong, Institute of Life Sciences, Shandong Provincial Key Laboratory for Distributed Computer Software Novel Technology, and Key Lab of Intelligent Information Processing, Jinan, 250358 China; 2 Shandong University of Science and Technology, Department of Electrical Engineering Information Technology, Jinan, 250031 China; 3 Shandong Provincial Hospital Affiliated to Shandong University, Department of Ophthalmology, Jinan, 250021 China; 4 Shandong Normal University, School of Psychology, Jinan, 250358 China; 5 University of North Carolina, Department of Radiology and BRIC, Chapel Hill, NC 27599 USA; 6 Korea University, Department of Brain and Cognitive Engineering, Seoul, 02841 Republic of Korea

## Abstract

Multispectral imaging (MSI) creates a series of en-face fundus spectral sections by leveraging an extensive range of discrete monochromatic light sources and allows for an examination of the retina’s early morphologic changes that are not generally visible with traditional fundus imaging modalities. An Ophthalmologist’s interpretation of MSI images is commonly conducted by qualitatively analyzing the spectral consistency between degenerated areas and normal ones, which characterizes the image variation across different spectra. Unfortunately, an ophthalmologist’s interpretation is practically difficult considering the fact that human perception is limited to the RGB color space, while an MSI sequence contains typically more than ten spectra. In this paper, we propose a method for measuring the spectral inconsistency of MSI images without supervision, which yields quantitative information indicating the pathological property of the tissue. Specifically, we define mathematically the spectral consistency as an existence of a pixel-specific latent feature vector and a spectrum-specific projection matrix, which can be used to reconstruct the representative features of pixels. The spectral inconsistency is then measured using the number of latent feature vectors required to reconstruct the representative features in practice. Experimental results from 54 MSI sequences show that our spectral inconsistency measurement is potentially invaluable for MSI-based ocular disease diagnosis.

## Introduction

**Figure 1:**
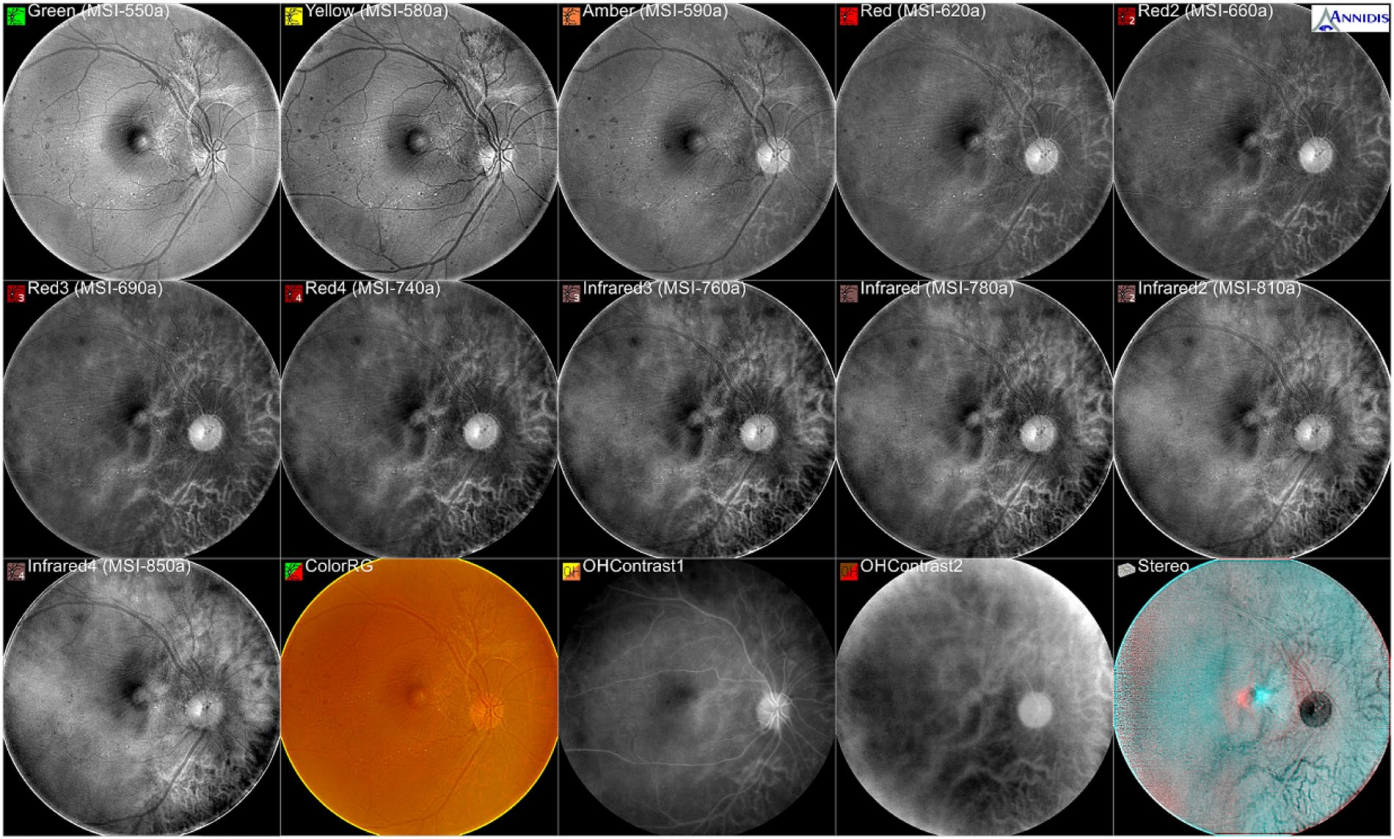
A collection of MSI images captured by Annidis RHA from a patient aged 60 and diagnosed with diabetic retinopathy. Ordered from left to right and from top to bottom, the first 11 sequential images are captured with short wavelengths of MSI-550, MSI-580, MSI-590, MSI-620, MSI-660, MSI-690, MSI-740, MSI-760, MSI-780, MSI-810 and MSI-850, respectively.

Multispectral imaging (MSI) is non-invasive computational photography that penetrates various features of physical objects within the field of view by leveraging a variety of spectral bands^1–3^. MSI involves light from frequencies not only within the visible light range but also from beyond (e.g. infrared), thus allowing extraction of additional information that is difficult for a human eye to capture. MSI has thus established itself as an important imaging tool in research areas of airborne mapping, astronomical imaging, dentistry, dermatology, histopathology and ophthalmology^4^. For ophthalmologists, MSI offers dissections and visualizations of the inner limiting membrane all the way to the choroid by taking a sequence of spectral images via MSI systems like the Retinal Health Assessment (RHA, Annidis Health System Corp., as shown in Fig. 1)^5–7^. As an advanced imaging modality, MSI enables ophthalmologists’ differentiation of and followup on a wide variety of complex eye conditions and diseases. Interpreting MSI images based on human visual inspection remains the reference standard in MSI applications. However, computer-based algorithms are attracting more and more research interests due to their ability to provide objective and automatic image assessments.

An Ophthalmologist’s diagnosis with MSI is commonly carried out by examining the spectral inconsistency of retinal degenerations compared with normal tissues, which reflects the absorbtion variations of light in different wavelengths. MSI differs from other retinal imaging instruments in that it exploits an extensive range of discrete monochromatic LED-sourced wavelengths (ranging from 550 nm to 950 nm) to create a collection of noninvasive *en-face* spectral sections, which highlight the anatomic and metabolic signatures throughout the thickness of retina and choroid. The long-wavelength light (beyond 600 nm) reveals properties of melanin while a slightly longer wavelength reflects liposfuscin^5^. In contrast, wavelenghths ranging from 533 nm to 850 nm are selectively absorbed by hemoglobin, melanin and macular pigments. An assessment of the spectral inconsistency allows ophthalmologist’s early detection of pathologies. For example, early changes of retinal pigment epithelium (RPE) of RPE disruption are observed as normal structures in short wavelengths and a slight structural variation in pigment pattern in the red (620 nm, 660 nm, 690 nm and 740 nm) and infrared (760 nm, 780 nm, 810 nm and 850 nm) spectral slices^4^.

Visual estimation of the MSI spectral inconsistency remains the reference standard for MSI-based diagnostic pathology, with which the ophthalmologist qualitatively assesses image variations across different spectra and compares these variation properties between different locations. However, automated and quantitative assessment of the spectral inconsistency is increasingly required along with elevated usage of MSI. Automated tools are potentially capable of providing more prognostic and predictive markers, which might enable ophthalmologists to assess the aggressiveness of a disease in its early stage or review its response to therapy. In addition, automated analysis is not aimed at replacing but rather assisting the ophthalmologists, to not only increase diagnostic precision and inter-observer reliability but also reduce labor costs.

In this paper, we propose a method for measuring MSI spectral inconsistency based on an outlier detection framework, which can be used to detect retinal degenerations and segment the corresponding deteriorated regions. Specifically, we measure spectral consistency by extracting the common spectral properties of normal tissues and specify degenerations as outliers that bear inconsistent spectral properties with normal tissues. Mathematically, we define spectral consistency as the fact that the representative features of any pixel in any spectrum can be reconstructed by projecting linearly a unique pixel-specific latent feature vector with a spectrum-specific projection matrix. In contrast, the reconstruction of a spectrally inconsistent pixel requires more than one latent feature vector. The proposed method is founded on a probabilistic Gaussian mixture model and designed to find a MAP (maximum a posteriori) estimate of the projection matrix and the assignment to the latent feature vector(s) via a stochastic expectation-maximization (SEM) algorithm. One unique property of this algorithm lies in the fact that the latent feature vectors do not need to be explicitly resolved, leading to a robust and fast estimation.

## Results

Our validation database is comprised of 54 MSI image sequences acquired by using an Annidis RHATM instrument (Annidis Health Systems Corp., Ottawa, Canada). These images are of oculus dexter (OD) or oculus sinister (OS) from 5 healthy subjects and 22 patients diagnosed with diabetic retinopathy, branch retinal vein occlusion or age-related macular degeneration. From the unhealthy images, microaneurysm, retinal hemorrhages, hard exudates, cotton wool spots, retinal thickening or macular edema were documented. These images are provided in the format of DICOM with a bit depth of 16 and in size of 208 × 2048. Each sequence bears 11 spectral slices captured with wavelengths of MSI-550, MSI-580, MSI-590, MSI-620, MSI-660, MSI-690, MSI-740, MSI-760, MSI-780, MSI-810 and MSI-850, which correspond to amber, green, infrared, red and yellow, respectively.

The spectral inconsistency measurement approach we present in this paper offers a score value for each pixel to indicate the probability (a value in [01]) of this pixel being degenerated. We carry out 3 experiments in order to validate the usefulness of the output of the proposed approach. First, we compare both qualitatively and quantitatively between the algorithm-measured spectral inconsistency and the manually delineated degenerated regions in the MSI sequence. Second, we show that the inclusion of spectral inconsistency as an additional feature can help boost the performance of the classification-based retinal degeneration segmentation algorithm. Third, we test the speed of and agreement between ophthalmologists in manual segmentation of retinal degenerations and compare between a case of using the spectral inconsistency as a guide and another case without using it.

To eliminate the spatial misalignment between MSI spectral slices, we employ the semidefinite programming-based joint alignment algorithm^8^ to register the sequential images in each MSI sequence. This algorithm solves a low-rank and semidefinite matrix that stores all pairwise-image feature mappings by minimizing the total amount of point-to-point matching cost via a convex optimization of a semidefinite programming formulation. It takes in a complete consideration of the information aggregated by all point-matching costs and enables the entire set of pairwise-image feature mappings to be solved simultaneously and near-optimally. This algorithm has been shown to be extremely effective in aligning sequential multimodel images.

### Agreement Between Spectral Inconsistency and Degenerated Retinal Regions

**Figure 2:**
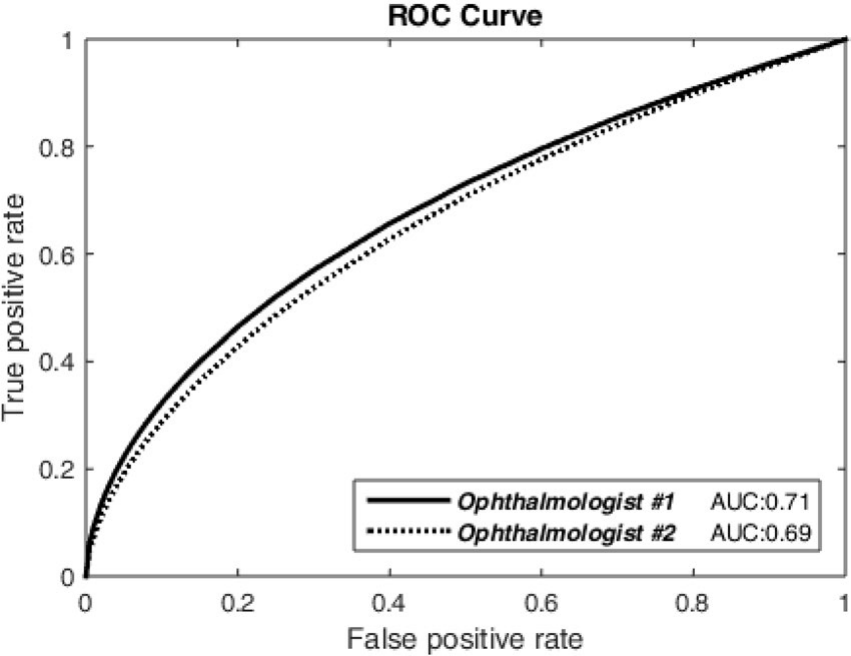
ROC curves generated by comparing the manual degeneration delineations from two ophthalmologists and our algorithm’s corresponding segmentations, which are obtained by establishing a threshold for the spectral inconsistency with different thresholds.

**Figure 3:**
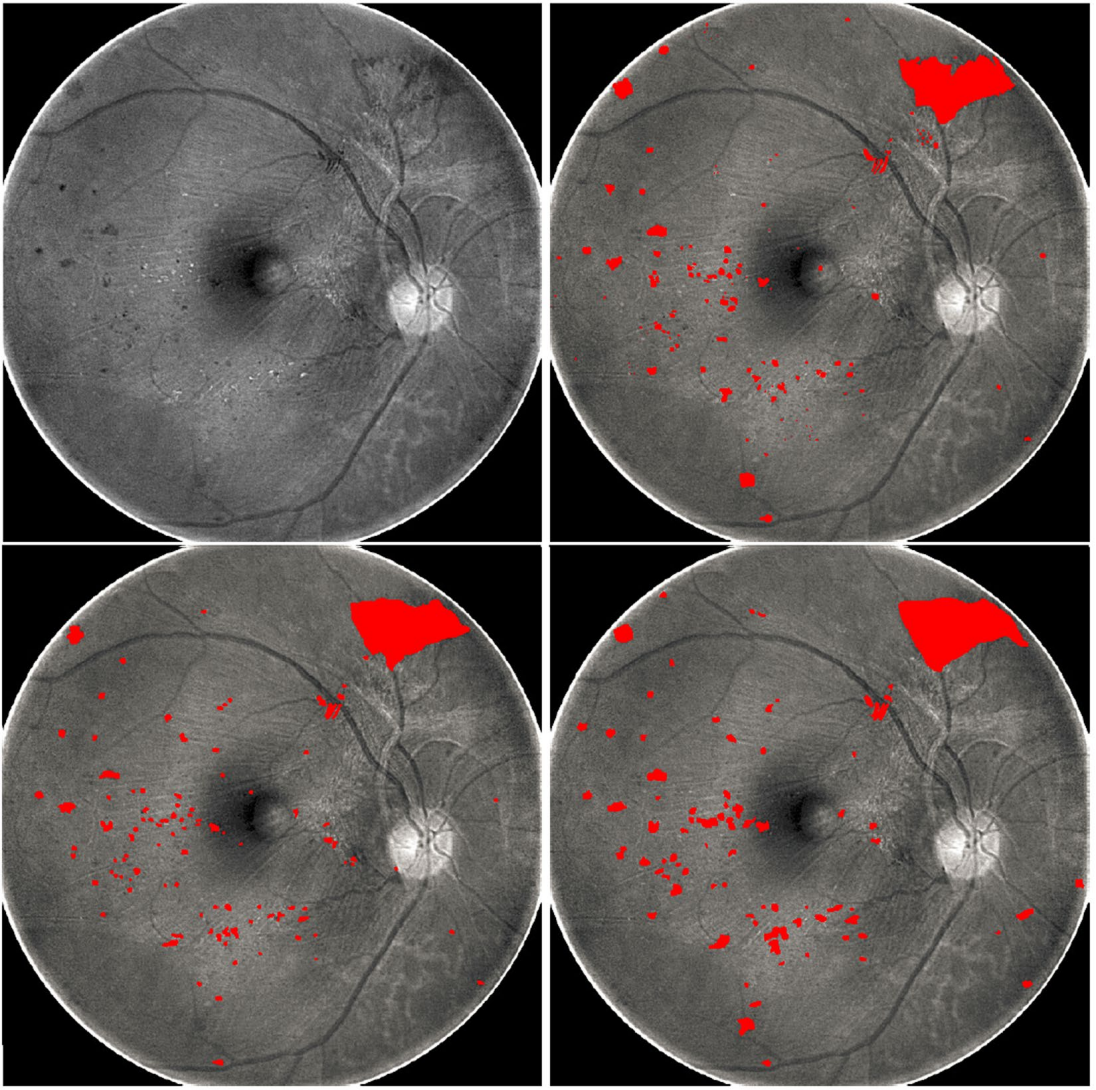
Manual degeneration delineations (lower two images) from two ophthalmologists and the automatic degeneration detection result (top-right) obtained with the best threshold on the spectral inconsistency measured by our algorithm, as indicated by the red color added in the spectral slice of MSI-550 (top-left) in Fig. 1.

To assess the agreement between the spectral inconsistency and the degenerated retinal regions, we randomly chose 34 MSI sequences (3 from healthy subjects and 31 from patients), and in each sequence from patients, two ophthalmologists manually drew the degenerated areas by carefully observing and comparing different spectral slices. We then ran our algorithm to measure the spectral inconsistency value for each pixel of each sequence. We then obtained a set of degenerative regions by establishing a threshold for the spectral inconsistency in a way that pixels with a value above this threshold are treated as degenerated and as healthy otherwise. By varying the threshold value, we plotted the receiver operating characteristic (ROC) curve using the ROCKIT algorithm^9^ and calculated the area under the curve (AUC) values, as shown by the results in Fig. 2. In addition to the AUC value, we also found that the best segmentation (as shown in Fig. 3) accuracy of our algorithm is 0.73 and 0.75 when treating the manual delineations of ophthalmologist #1 and #2 as the gold standard, respectively. From Fig. 3, we can see that microaneurysm, retinal hemorrhages and hard exudates can be observed from this subject, and our algorithm detected them successfully.

### Classification-based Degeneration Recognition by Using Spectral Inconsistency

**Figure 4:**
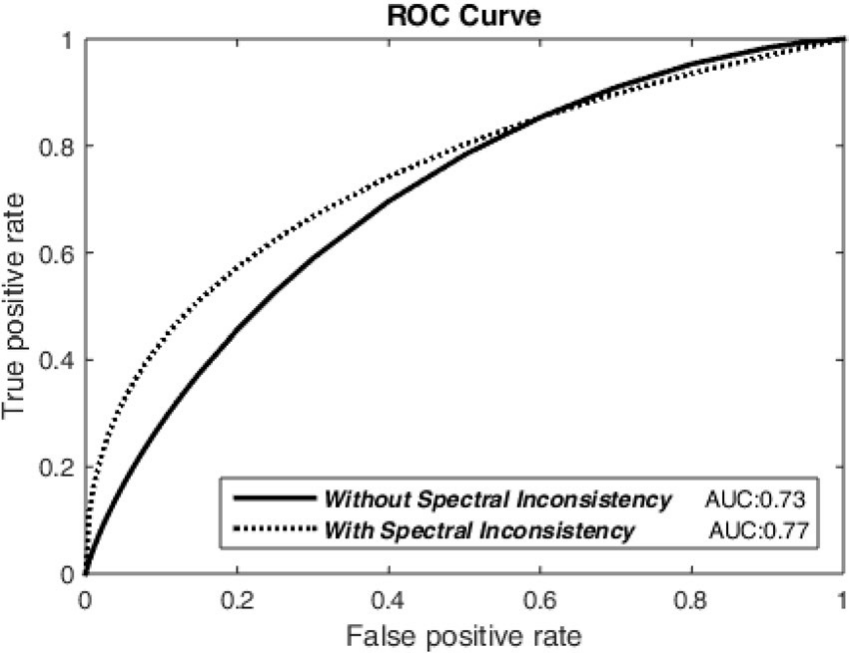
ROC curves measuring the performance of the SVM classifier in distinguishing the degenerated pixels and the normal ones, by including or not including the spectral inconsistency as an additional feature, respectively.

We trained a SVM binary classifier^10^ with a Gaussian kernel to distinguish normal pixels from the degenerated ones. The classifier treats all spectral values of each pixel as its features. A leave-one-out cross-validation strategy is exploited, which involves treating each of the 34 MSI sequences as the validation set and the remaining as the training set and repeating on all these ways to cut the original set into validation and training. We then consider the spectral inconsistency measured by our algorithm as an additional feature of the SVM classifier in order to validate its value in this per-pixel classification. The AUC values obtained without/with the spectral inconsistency are 0.73/0.77 and 0.74/0.76 when treating ophthalmologist #1 (as shown in Fig. 4) and ophthalmologist #2 as the gold-standard, respectively. In addition, we found that the best accuracies without/with using the spectral inconsistency are 0.79/0.84 and 0.79/0.83 when treating ophthalmologist #1 and ophthalmologist #2 as the gold-standard, respectively.

### Manual Retinal Degeneration Segmentation Guided by Spectral Inconsistency

The spectral inconsistency measured by our algorithm is also invaluable due to its guidance on ophthalmologist’s diagnosis. This guidance is actually founded on the fact that the spectral inconsistency indicates the probability of the pixel being degenerated. By observing the spectral inconsistency map, ophthalmologists may locate and recognize the details of the degenerated area more easily and quickly.

To validate this benefit from the spectral inconsistency, the two ophthalmologists manually outlined the degeneration by observing all the MSI spectral slices and the generated spectral inconsistency map for the remaining 20 MSI sequences. The average time (averaged over these two ophthalmologists) spent on each sequence is reduced from approximately 25 minutes on the 34 sequences (for which the spectral inconsistency was not involved) to approximately 14 minutes on the 20 sequences (for which the spectral inconsistency was used). In addition, we found that the Dice’s coefficient (averaged over these two ophthalmologists), which measures the overlapping ratio between the two ophthalmologists’ segmentation, is improved from 0.84 (generated without the spectral inconsistency) to 0.91 (generated with the spectral inconsistency).

## Discussion

MSI employs multiple wavelengths of light sources to capture images of the retina and choroid and provides the ophthalmologists with the identification, interpretation, diagnosis and management of ocular pathology via spectral dissections. MSI represents a major advance in ocular diagnostics^4^ because the carefully selected spectral bands used by MSI are targeted to the clinically relevant structures and metabolic characteristics, particularly the ocular chromophores melanin and hemoglobin, as well as the fluorophore lipofuscin. MSI allows for an isolation, identification and interpretation of the early and subtle ocular pathologies that are often difficult when using a traditional instrument.

However, visual inspection-based diagnosis with MSI is absolutely not trivial although it remains the reference standard. In the process of visual inspection of the MSI images, the ophthalmologists observe and compare the spectral characteristics across different spectra in order to measure qualitatively the spectral inconsistency. This is rather difficult considering the fact that the number of spectral slices in an MSI sequence may be more than 10 (as in the data set we are using in this paper).

In this paper, we propose a generative model for quantitatively measuring the MSI spectral inconsistency, which is validated to be able to reveal retinal degenerations. This model is built on our proposed mathematical definition of spectral consistency, which considers a pixel to be spectrally consistent when its representative features at any spectrum can be reconstructed by a linear projection of a single latent feature vector with a spectrum-specific projection matrix. With this definition, spectral inconsistency means a requirement of more than one latent feature vectors in the linear reconstruction. One key advantage of the proposed model is that the latent feature vectors (which play an import role in measuring the spectral inconsistency) are free from being inferred explicitly, which makes our algorithm robust and fast.

We have several findings from the experimental results described in the last section. First, segmentation of retinal degenerations by establishing a threshold for the spectral inconsistency measured using our algorithm complies well with manual segmentation of experienced ophthalmologists. This also means that the spectral inconsistency is potentially an invaluable indicator of retinal degenerations. Second, the spectral inconsistency measurement can help boost the performance of a classification algorithm designed for distinguishing the degenerated pixels from the normal ones and therefore can be treated as a new biomarker, which is potentially useful for early detection of pathologies. Third, the spectral inconsistency measurement can also offer a guidance for the ophthalmologists on recognizing pathological areas from MSI images, which reduces human effort and increases human speed in analyzing the sequential slices of MSI.

Disregarding the concrete generative model (to be detailed in next section) for measuring the spectral inconsistency from MSI, we can explain the mystery of why the proposed spectral inconsistency measurement can help to recognize the pathological areas from MSI based on an application of a similar mathematical model with multi-view anomaly detection^11^. Anomaly detection means identifying outlier observations, which do not conform to an expected pattern in a dataset. Unsupervised anomaly detection assumes that the majorities of the observations in the provided dataset are normal and looks for observations that seem to least fit to the remainder (i.e., outliers) of the dataset. In our spectral inconsistency measurement based on the proposed generative model, we assume that most pixels in MSI are normal, and then focus our efforts on searching for the potential outlier (i.e., degenerated) pixels whose spectral properties are different from the normal ones. The resulted spectral inconsistency is therefore associated with the probability of being pathological. This unsupervised identification of retinal pathologies is potentially invaluable in early diagnosis and easier differentiation of occult or overlapping pathologies.

## Methods

Our method is based on a basic assumption that normal retinal tissues are consistent over certain spectral properties, whereas retinal degenerations are inconsistent. We propose a generative model approach for extracting the consistent spectral properties of normal tissues and measuring the spectral inconsistency of each retinal pixel, with which we can detect and segment the regions bearing degenerative changes by establishing a threshold for the spectral inconsistency. Specifically, we define spectral consistency as the fact that the representative features at any spectrum of a pixel can be reconstructed by a linear projection of a single latent feature vector with a spectrum-specific projection matrix. In contrast, the reconstruction of spectrally inconsistent pixels requires more than one latent feature vectors. As a key advantage of the proposed model, the latent feature vector are not needed to be explicitly inferred, which makes the algorithm robust and fast.

### Probabilistic Model for Measuring Spectral Inconsistency

We are given a sequence of *s* retinal MSI spectral slices, each with *n* pixels. We then have a data matrix ${\bf{X}}=[{{\bf{x}}}_{1},\cdots ,{{\bf{x}}}_{n}]\in {{\mathbb{R}}}^{d\times n}$, where ${{\bf{x}}}_{i}\in {{\mathbb{R}}}^{d}$ denotes a vector of features extracted from all spectral slices at pixel *i*. Supposing that there are *d*_*j*_ features extracted from the *j*th spectral slice, we then have $d={\sum }_{j=1}^{s}{d}_{j}$. We use **x**_*ij*_ to represent the vector of features extracted at pixel *i* and from the *j*th spectral band. Our goal is then to specify degenerative pixels that bear inconsistent observation features across different spectral slices compared with normal pixels.

We define mathematically that a pixel *i* is spectrally consistent if its representative features *x*_*ij*_ at any spectrum *j* can be reconstructed by a linear projection of a single latent feature vector ${z}_{i}\in {{\mathbb{R}}}^{l}$ with a spectrum-specific projection matrix ${{\bf{P}}}_{j}\in {{\mathbb{R}}}^{{d}_{j}\times l}$, i.e., ${{\bf{x}}}_{ij}={{\bf{P}}}_{j}{{\bf{z}}}_{i}$. We can see from this definition that **z**_*i*_ is shared in the reconstruction of all spectra for a spectrally consistent pixel *i*. At the same time, we assume **P**_*j*_ is shared by all pixels. In contrast, for a pixel *i* that is spectrally inconsistent, different spectra might be reconstructed with different latent feature vectors, which are denoted by ${{\bf{Z}}}_{i}=\{{{\bf{z}}}_{im},m\in {{\mathbb{Z}}}^{+}\}$, where ${{\mathbb{Z}}}^{+}$ represents the set of positive integers.

In the probabilistic model introduced in this paper, the probability of the data **x**_*ij*_ for the *i*th pixel and *j*th spectrum is calculated with the following Gaussian mixture model

1 $$p({x}_{ij}|{{\bf{Z}}}_{i},{{\bf{P}}}_{j},{{\bf{W}}}_{i},{\sigma }^{-1}{\bf{I}})=\sum _{m\in {{\mathbb{Z}}}^{+}}{w}_{im}{\bf{N}}({{\bf{x}}}_{ij}|{{\bf{P}}}_{j}{{\bf{z}}}_{im},{\bf{I}}/\sigma ),$$

where **N(x|**μ, Σ) denotes a Gaussian distribution with mean *μ* and covariance matrix Σ, ${{\bf{W}}}_{i}=\{{{\bf{w}}}_{im}|m\in {{\mathbb{Z}}}^{+}\}$ are the mixture weights, *σ* is a precision parameter and **I** means an identity matrix. We generate the mixture weights *w*_*m*_ with a Dirichlet process, which is accomplished by a stick-breaking process^12^ with a concentration parameter *γ*. At the same time, we assume that *σ* and the latent feature vector **z**_*im*_ are drawn from a Gamma distribution Gamma *(α*, *β*) and a Gaussian distribution **N**(**0**, **I**/*(σr*)), respectively.

To measure the spectral inconsistency of retinal MSI based on the probabilistic model in Eq (1), we employ the SEM algorithm^11^. This algorithm is an iterative method to find a MAP estimate of the projection matrix {**P**_*j*_} and a latent vector assignment ${\bf{S}}=\{{s}_{ij}\in {{\mathbb{Z}}}^{+}\}$ where *s*_*ij*_ indexes the latent vector in **Z**_*i*_ used for reconstructing **x**_*ij*_. One strength of this algorithm lies in its property of being free from the requirement of an estimation of the latent feature vectors **Z**_*i*_, mixture weights **W**_*i*_ and precision parameter *σ* because they can be marginalized out analytically.

#### E-Step

The E-step of the SEM algorithm samples a latent vector assignment value for any pixel *i* and spectrum *j* while fixing the projection matrix **P** with the following probability function

2 $$p({s}_{ij}=m|{\bf{X}},{{\bf{S}}}_{-ij},{\bf{P}},\alpha ,\beta ,r,\gamma )\propto \frac{p({s}_{ij}=m,{{\bf{S}}}_{-ij}|\gamma )}{p({{\bf{S}}}_{-ij}|\gamma )}\cdot \frac{p({\bf{X}}|{{s}}_{ij}=m,{{\bf{S}}}_{-ij},{\bf{P}},\alpha ,\beta ,r)}{p({{\bf{X}}}_{-ij}|{{\bf{S}}}_{-ij},{\bf{P}},\alpha ,\beta ,r)}$$

where −*ij* means the related set excluding pixel *i* and spectrum *j*.

Mathematical deductions of Eq. (2) can be conducted for two cases of *s*_*ij*_: 1) sampling from the existing latent feature vectors and 2) introducing a new latent feature vector. With a set of mathematical deductions^11^, Eq. (2) can be represented as the followings for the former case:

3 $$p({s}_{ij}=m|{\bf{X}},{S}_{-ij},{\bf{P}},\alpha ,\beta ,r,\gamma )\propto \frac{{N}_{im-j}}{D-1+\gamma }\frac{{B}_{-ij}^{{A}_{-ij}}}{{B}_{{s}_{ij}=m}^{A}}\frac{{\rm{\Gamma }}(A)}{{\rm{\Gamma }}({A}_{-ij})}\frac{|{{\bf{C}}}_{m,{s}_{ij}=m}{|}^{\mathrm{1/2}}}{|{{\bf{C}}}_{m-ij}{|}^{\mathrm{1/2}}}$$

where *N*_*im*−*j*_ denotes the number of spectra assigned to the *m*th latent feature vector without considering the *j*th spectrum, and −*ij* means the related values computed by excluding pixel *i* and spectrum *j* In Eq (3), *A* and *B* are calculated by

4 $$A=\alpha +\frac{n\sum _{j=1}^{s}{d}_{j}}{2}$$

and

5 $$B=\beta +\mathrm{1/2}(\sum _{i=1}^{n}\sum _{j=1}^{s}{{\bf{x}}}_{ij}^{^{\prime} }{{\bf{x}}}_{ij}-\sum _{i=1}^{n}\sum _{m=1}^{{M}_{i}}{\mu }_{im}^{^{\prime} }{{\bf{C}}}_{im}^{-1}{\mu }_{im})$$

where *M*_*i*_ denotes the number of latent feature vectors created for pixel *i*,

6 $${\mu }_{im}={{\bf{C}}}_{im}\sum _{\{j\in \mathrm{\{1,2,}\cdots ,s\}|{s}_{ij}=m\}}{{\bf{P}}}_{j}^{^{\prime} }{{\bf{x}}}_{ij}\quad {\rm{and}}\quad {{\bf{C}}}_{im}^{-1}=\sum _{\{j\in \mathrm{\{1,2,}\cdots ,s\}|{s}_{ij}=m\}}{{\bf{P}}}_{j}^{^{\prime} }{{\bf{P}}}_{j}+r{\bf{I}}\mathrm{.}$$

In Eq. (3),

7 $${B}_{{s}_{ij}=m}={B}_{-ij}+\mathrm{1/2}({{\bf{x}}}_{ij}^{^{\prime} }{{\bf{x}}}_{ij}+{\mu }_{im-ij}^{^{\prime} }{{\bf{C}}}_{im-ij}^{-1}{\mu }_{im-ij}-{\mu }_{im,{s}_{ij}=m}^{^{\prime} }{{\bf{C}}}_{im,{s}_{ij}=m}^{-1}{\mu }_{im,{s}_{ij}=m})$$

where

8 $${\mu }_{im-ij}={{\bf{C}}}_{im,{s}_{ij}=m}({{\bf{P}}}_{j}^{^{\prime} }{{\bf{x}}}_{ij}+{{\bf{C}}}_{im-i{j}^{-1}}{\mu }_{im-ij})\quad {\rm{and}}\quad {{\bf{C}}}_{im,{s}_{ij}=m}^{-1}={{\bf{P}}}_{j}^{^{\prime} }{{\bf{P}}}_{j}+{{\bf{C}}}_{im-ij}^{-1}\mathrm{.}$$

When a new latent feature vector is needed in order to determine *s*_*ij*_, Eq. (2) is expressed as

9 $$p({s}_{ij}=m|{\bf{X}},{{\bf{S}}}_{-ij},{\bf{P}},\alpha ,\beta ,r,\gamma )\propto \frac{{r}^{l\mathrm{/2}}\gamma }{D-1+\gamma }\frac{{B}_{-ij}^{{A}_{-ij}}}{{B}_{{s}_{ij}=m}^{A}}\frac{{\rm{\Gamma }}(A)}{{\rm{\Gamma }}({A}_{-ij})}\frac{|{{\bf{C}}}_{m,{s}_{ij}=m}{|}^{\mathrm{1/2}}}{|{{\bf{C}}}_{m-ij}{|}^{\mathrm{1/2}}}\mathrm{.}$$

#### M-Step

The M-step of the SEM algorithm estimates the projection matrix **P** while fixing the latent feature assignment **S** with the following estimation equation, which is obtained by maximizing the logarithm of the joint likelihood of **X** and **S** given **P**, *α*, *β*, *r* and *γ* (here we omit the deduction details for economy).

10 $${{\bf{P}}}_{j}=\frac{A}{B}(\sum _{i\mathrm{=1}}^{n}{{\bf{x}}}_{ij}{\mu }_{i{s}_{ij}}^{^{\prime} }){(\sum _{i\mathrm{=1}}^{n}\sum _{m\mathrm{=1}}^{{M}_{i}}{{\bf{C}}}_{im}+\frac{A}{B}\sum _{i\mathrm{=1}}^{n}{\mu }_{m{s}_{ij}}{\mu }_{m{s}_{ij}}^{^{\prime} })}^{-1}$$

The SEM algorithm iterates the E-step and the M-step, which samples *s*_*ij*_ for each pixel *i* and spectrum *j* using Eq. (2) and estimates the projection matrix **P**_*j*_ for each spectrum *j*.

#### Measuring Spectral Inconsistency

For each pixel *i*, we measure its spectral inconsistency *ψ*_*i*_ by computing the average probability of the event *M*_*i*_ > 1 over all iterations of the SEM algorithm with the following equation

11 $${\psi }_{i}=\frac{1}{T}\sum _{t=1}^{T}{{\mathbb{1}}}_{{M}_{i} > 1}$$

where ${\mathbb{1}}$ is the indicator function which takes a value of 1 if the condition *M*_*i*_ > 1 is satisfied and 0 otherwise. Obviously, the condition *M*_*i*_ > 1 can be evaluated by using the estimations of *s*_*ij*_ in the E-Step of the SEM algorithm.

#### Specifying Representative Features and Setting Parameters

The representative features are extracted to describe the local image pattern around each pixel and specified as the image gradient orientation^13^ rather than the image intensity. As shown in several works^14, 15^, image gradient orientations can boost the performance of image classification, multi-output regression and subspace learning. Specifically, we compute the image gradients and the corresponding gradient orientation at each pixel of each spectral slice. We then treat the vector formed by concatenating all gradient orientations with a local window (in a size such as 7 × 7) as the representative feature vector of this pixel.

We terminated the algorithm after 400 iterations and empirically set the parameters *γ* = 1.2, *α* = 1 and *β* = 1. We specified the length *l* of the latent feature vector with a cross-validation process. Specifically, we compared the manually drawn degenerative regions of retina with the algorithm’s results generated with different *l* values and then chose the smallest number of *l* with the best accuracy.

### Data availability statement

The datasets analysed during the current study are available from the corresponding author on reasonable request.
